# An Overview of Machine Learning Applications in Sports Injury Prediction

**DOI:** 10.7759/cureus.46170

**Published:** 2023-09-28

**Authors:** Alfred Amendolara, Devin Pfister, Marina Settelmayer, Mujtaba Shah, Veronica Wu, Sean Donnelly, Brooke Johnston, Race Peterson, David Sant, John Kriak, Kyle Bills

**Affiliations:** 1 Federated Department of Biology, New Jersey Institute of Technology, Newark, USA; 2 Department of Biomedical Sciences, Noorda College of Osteopathic Medicine, Provo, USA

**Keywords:** sports injury prevention, modeling, injury prediction, sports medicine, ai and machine learning

## Abstract

Use injuries, i.e., injuries caused by repetitive strain on the body, represent a serious problem in athletics that has traditionally relied on historic datasets and human experience for prevention. Existing methodologies have been frustratingly slow at developing higher precision prevention practices. Technological advancements have permitted the emergence of artificial intelligence and machine learning (ML) as promising toolsets to enhance both injury mitigation and rehabilitation protocols. This article provides a comprehensive overview of recent advances in ML techniques as they have been applied to sports injury prediction and prevention. A comprehensive literature review was conducted searching PubMed/Medline, Institute of Electrical and Electronics Engineers (IEEE)/Institute of Engineering and Technology (IET), and ScienceDirect. Ovid Discovery and Google Scholar were used to provide additional aggregate results and a grey literature search. A focus was placed on papers published from 2017 to 2022. Algorithms of interest were limited to K-Nearest Neighbor (KNN), K-means, decision tree, random forest, gradient boosting and AdaBoost, and neural networks. A total of 42 original research papers were included, and their results were summarized. We conclude that given the current lack of open source, uniform data sets, as well as a reliance on dated regression models, no strong conclusions about the real-world efficacy of ML as it applies to sports injury prediction can be made. However, it is suggested that addressing these two issues will allow powerful, novel ML architectures to be deployed, thus rapidly advancing the state of this field, and providing validated clinical tools.

## Introduction and background

Machine learning (ML) is a complex discipline broadly defined as the creation of a computer system able to experientially learn and adapt without explicit instructions to generate predictive analytics [1,2]. As computational resources have continued to increase, ML application and implementation in various fields has grown, sports medicine included. The assessment, mitigation, and prevention of injury is of primary importance as injuries are ubiquitous and may result in severe physical, emotional, and financial consequences, especially at the professional level. To elucidate the complex factors contributing to athlete injuries and to enable greater predictive precision, a variety of ML models have been proposed in the literature [3-6].

As computational technologies advance, larger and more complex ML algorithms, including application of previously theoretical techniques, are possible. It is therefore useful to periodically compile and review literature that has been, or may be, applied to injury prediction and prevention. Additionally, though recent literature reviews explore niche aspects of this field, limitations exist: articles are written from the perspective of data mining and without interest in recency [5], are sports-specific [7-9], are limited in scope [3,4,10], or are focused on team sports only [6]. We seek to provide a comprehensive overview of the state of ML in sports injury across many sports using a broad selection of algorithms. To provide a basis for the exploration of novel ML models and methodologies, algorithms have been categorized based on function, limitations, and current or potential implementation to sports medicine.

## Review

Methods

A comprehensive literature review was conducted. Ovid Discovery search and Google Scholar provided compiled results from many databases. PubMed/Medline, Institute of Electrical and Electronics Engineers (IEEE)/Institute of Engineering and Technology (IET), and ScienceDirect were accessed individually. A focus was placed on papers published from 2017 to 2022. Algorithms were selected based on a preliminary literature review and included K-Nearest Neighbor (KNN), K-means, decision tree, random forest, gradient boosting and AdaBoost, and neural networks (NNs). Search terms were “algorithm name” + “sport” + “injury” for each algorithm, e.g., “neural network” + “sport” + “injury”. An attempt was made to include variations in algorithm name and abbreviation. Papers concerning prediction and analysis of sports injuries were included. Any papers that could not be accessed or were not available in English were excluded. Forty-two original research papers and eight review articles were selected based on the criteria described. Of note, we excluded papers primarily relying on linear or logistic regression as we feel these algorithms do not represent the cutting edge of predictive analysis and have been addressed elsewhere in the literature. This article was previously posted to the SportRxiv preprint server on November 16, 2022.

Results

Results of the comprehensive literature review are summarized below. Papers were sorted into these sections based on the algorithm tested. When more than one algorithm was explored, papers were included in the section with the most effective algorithm and in sections with algorithms that were nearly as successful where appropriate. Due to variable study design, and often disparate aims, no attempt has been made to directly compare or otherwise aggregate results quantitatively. Instead, we present overall trends in the Discussion. Likewise, shortcomings or pitfalls have been addressed in the Discussion section. Note that due to the diversity of neural network implementations, papers pertaining to neural networks have been further subdivided.

KNN

In sports medicine, special sensors like accelerometers, gyroscopes, infrared sensors, and magnetometers can be attached to athletes to collect data. Using data collected from different body parts of athletes, KNN may analyze behaviors for athletes in unique sporting events. With this recognition model, patterns predisposing to injury can be determined, allowing for potential injury prevention [11]. In addition to their general use as comparison algorithms, a 2018 paper applied KNN as part of a larger model, including both K-means and support vector machine (SVM), for injury prediction [12].

K-Means

In 2020, a study by Dingenen et al. used K-means to establish that runners with the same injuries could be clustered into two different subgroups with a mean silhouette coefficient of 0.53 [13]. These subgroups were used to illustrate variable kinematic causes of running-related injury. K-means was also used by Ibáñez et al. in 2022 as a data separation technique for grouping women’s basketball players into first and second divisions. This study effectively used K-means to analyze thresholds of deceleration, acceleration, speed, and impact on the players and determined a difference between the first and second divisions [14]. These so-called divisions were proposed to aid in personalization of training to prevent injuries and improve performance. As seen in these recent articles, and likely due to its simplicity and familiarity, K-means remains effective when applied to traditional clustering problems and may be suited to exploring injury risk factors or player characteristics.

Support Vector Machines

For sports-specific applications, SVMs have been trained using modifiable metrics such as training load, performance techniques, psychological and neuromuscular assessments, and non-modifiable metrics such as anthropometric measurements, previous injury history, and genetic markers to accurately predict future injuries [4,15]. The identification of injury risk factors such as these allows coaches and medical personnel to modify training loads, regiments, and techniques to potentially prevent future injuries [6]. For example, a 2018 paper by Ruddy et al. used a number of ML algorithms, including SVM, to assess risk factors identified in hamstring strain injuries [16]. In another 2018 paper by Carey et al., also exploring hamstring injury prediction and risk factors, SVM benefited substantially from data pre-processing, although it was ultimately outperformed by simple logistic regression [17]. Using non-physiological data, a 2017 paper predicting in-game injuries in Major League Soccer found that SVMs were the most accurate of several tested algorithms, including logistic regression, multilayer perceptron, and random forest [18]. However, in the recent literature, including two 2021 papers comparing efficacy of ML algorithms, SVMs have proven less effective than other algorithms [19,20]. Despite this, SVMs may still be valuable given their suitability for predicting high-dimensionality data sets, especially when combined with other techniques, as in a 2022 paper by Wang et al. predicting triple jump injury [21].

Decision Tree

Modern evolutions of the classic decision tree algorithm have been broadly applied in recent years. In 2018, Connaboy et al. used decision trees built with chi-squared automatic interaction detection (CHAID) to analyze factors contributing to lower extremity injury in military personnel. Using their model, the authors identified several factors leading to increased injury risk over a 365-day period [22]. Using a classification and regression tree (CART), Mendonça et al. investigated associations between various risk factors and patellar tendinopathy in volleyball and basketball players [23]. A 2021 paper by Kolodziej et al. applied a CART decision tree to predict youth soccer injuries, achieving a sensitivity of 0.73 and a specificity of 0.91 [24]. Another 2021 paper by Ruiz-Pérez et al. attempted to reproduce a 2020 model by Rommers et al., which used field data collected via GPS. While they favorably compared C4.5 decision trees with several modeling approaches including KNN, SVM, and ADTree, they did not use the same algorithm as Rommers et al. and did not achieve comparable performance (area under the receiver operating characteristic [ROC] curve, or AUC, 0.767 vs. 0.850) [25,26]. Contrary to these relatively promising results, Rossi et al. found that decision trees, although outperforming comparison algorithms, were not able to achieve a precision greater than 50% when forecasting soccer injuries [27]. Decision trees undoubtedly have a place in sports injury prediction, though their performance varies with data and model structure. Additionally, they can lack generalizability and overfit during training, thus limiting their accuracy [28].

Random Forest

Random forest models have been applied to injury prediction with mixed success. In a study of sports-related dental injuries in children, random forest algorithms had a slightly higher prediction accuracy when compared to the traditional regression methods [29]. A 2020 paper sought to address inconsistency in predictive performance by identifying key risk factors prior to training of the model. They were able to achieve an AUC of 0.79 [30]. In a 2022 paper, a random forest model was built and achieved similar performance with an AUC of 0.72 [31]. In an investigation of paralympic swimmers classifying participants with and without brain injury to determine eligibility, random forests successfully classified 96% of the 51 participants [32]. Contrary to these studies, a 2021 paper found that random forests predicted ankle injuries in young athletes with similar performance to a logistic regression (ROC 0.63 vs. 0.65, respectively) [33]. With proper application and unbiased feature selection, random forest models may be tuned to outperform existing classification methods, though they are sensitive to variations in data sets.

Gradient Boosting and AdaBoost Neural Networks

Gradient boosting regularly outperforms baseline regression and various ML algorithms including decision tree and SVM for certain classification problems [34-39]. Nicholson et al. found gradient boosting to be the most effective of several algorithms in assessing elbow valgus torque and shoulder distraction force in 168 high school and college pitchers [37]. Remarkably, a 2019 study predicting skier injuries found that gradient boosting produced a 0.25 increase in accuracy over logistic regression with an AUC of 0.76 versus 0.52 [34]. Hecksteden et al., in a 2022 prospective observation cohort study, also found that gradient boosting performed better than comparison algorithms when forecasting non-contact time-loss injuries in 88 soccer players [38].

Expanding beyond standard gradient boosting, a 2022 study used XGBoost (extreme gradient boosting) to predict post-concussion injuries in 74 college football players with an accuracy of 91.9% [40]. Rommers et al. in a 2020 paper also used XGBoost, this time predicting injuries in 734 youth soccer players with a precision and recall of 84% and 83%, respectively. The authors were also able to classify injuries as either overuse or acute with a precision and recall of 82% [26]. Additionally, a recent retrospective review used an XGBoost model to explore the relationship between biomechanics and self-reported athlete injury [41]. Notably, only one recent paper was found to use AdaBoost, a 2022 study predicting injury in CrossFit practitioners. AdaBoost was found to perform better overall than comparison algorithms with an AUC of 77.93% [36].

A 2018 study by López-Valenciano et al. found that a modified boosting algorithm called SMOTEBoost (synthetic minority oversampling technique) was able to predict musculoskeletal injuries in 132 football and handball players with an AUC of 0.747, a true positive rate of 65.9%, and a true negative rate of 79.1% [35]. Another similar algorithm called SmooteBoostM1 was used to predict hamstring injuries in professional soccer players, producing a model with an AUC of 0.837 [42]. Overall, gradient boosting, including the earlier AdaBoost and other modified boosting algorithms, represents a pronounced upgrade over classic logistic regression as well as ML algorithms such as decision tree, KNN, SVM, and multilayer perceptron when applied to the limited-class classification problem presented by predicting sports injury.

Convolutional Neural Networks

Kautz et al., in their 2017 work, used a convolutional neural network (CNN) to analyze wearable sensor data and allow for automated player monitoring in beach volleyball players. Compared to algorithms including SVM, KNN, Gaussian, and decision tree, the CNN provided significantly increased classification accuracy [43]. Pappalardo et al. developed a CNN to analyze multivariate time series extracted from electronic performance and tracking systems worn by professional soccer players. Their approach allowed for automated feature extraction, an advantage over more traditional time series analysis. Additionally, they were able to develop an injury forecaster that was explainable, which is a necessity for a deployable, real-world model [44]. Similarly, Chen et al. describe a process of converting time series data acquired from player-worn sensors to two-dimensional images for analysis using a CNN. Notably, they validate using only acceleration data from a single sensor and were able to achieve acceptable levels of accuracy in classification [11]. Song et al. in their 2020 study developed an optimized-CNN to predict and assess injuries in volleyball players. Using multidimensional sports data, they found that their algorithm was more accurate than comparison algorithms. Additionally, they described a framework for cloud-based deployment and integration with Internet of Things [45]. Ma and Pang in a 2019 paper also proposed a CNN for analysis of sports data using a real-time cloud-based system and Internet of Things [46]. Ghazi et al. in a 2021 paper described the use of CNN to estimate peak maximal principal strain in traumatic head injuries. Using data from the National Football League, they were able to achieve >90% accuracy in the prediction of concussion versus non-concussion [47].

Long Short-Term Memory Neural Networks

While long short-term memory (LSTM) nodes are primarily used for time series analysis, they may be combined with other algorithms to provide an advantage in prediction and classification problems because of their unique nature. In 2021, Meng et al. combined CNN with LSTM to allow for reliable analysis of two-dimensional data by the LSTM nodes. Using images of professional athletes, they were able to achieve 97.0% classification accuracy for risk stratification broken into no risk, low risk, medium risk, and high risk of injury. The model achieved a sensitivity of 95.70% and a specificity of 97.54% [19]. A combined architecture model such as this may ultimately yield more accurate algorithms.

Deep Gaussian Covariance Neural Networks

A 2022 paper by Rahlf et al. outlined a prospective study protocol using a deep Gaussian covariance network to analyze the relationship between internal and external factors contributing to runner injury. Recruitment for this study was ongoing at the time of publication [48]. This promises to provide real-world data on predictive performance of a neural network.

Radial Basis Function Neural Networks

In a 2021 study, Xiang applied a radial basis function (RBF)-based neural network to injury predictions. They stratified injury risk and validated using questionnaires sent to expert coaches [49]. Another 2021 paper proposed a similar RBF-based neural network to predict sports injuries. Injury risk was stratified into low risk, at risk, and high risk [50]. Notably, the author looked to determine which factors may contribute most to injury risk. Despite their novel premise, both papers lack robust validation or large data sets and are largely methodological.

Fuzzy and Grey Neural Network

A 2021 paper by Wang and Yang described the use of a fuzzy neural network to evaluate the degree of injury in sports. They found that the fuzzy neural network outperformed Bayesian and Lagrange models. However, this was a theoretical proposal using simulated data [51]. Another 2021 paper by Zhang et al. proposed a grey neural network that inputs the results of n-grey models into a neural network for final prediction. This too was a theoretical algorithm tested and validated with simulation data [52]. Despite their lack of real-world application, both papers present intriguing possibilities for integrating fuzzy and grey theory as a method of dealing with the inherent variability in sports injury data.

Table 1 summarizes key strengths and weaknesses based on the surveyed literature, along with the number of articles investigating each algorithm. Further details are provided in the Discussion section.

**Table 1:** Summary of findings Strengths and weaknesses of each algorithm have been presented, along with the number of papers included in this survey. Note that some studies have been counted in more than one category.

Algorithm	Number of studies	Strengths	Weaknesses	References
K-nearest neighbor	2	Simple to implement, unsupervised.	Sample size and data set size limitations, may be less accurate than other techniques, struggles with high-dimensionality data.	[11,12]
K-means	2	Simple to implement, unsupervised, useful for blind feature extraction and data exploration.	Better suited to initial data exploration than final classification when compared to other algorithms.	[13,14]
Support vector machines	9	Commonly integrated into ensemble models, increasing accuracy, able to handle high-dimensionality data.	Mixed success reported in the literature.	[4,6,15-21]
Decision tree	7	Reasonable accuracy combined with transparent decision making.	Struggles with high-dimensionality data.	[22-28]
Random forest	5	More accurate than decision trees while retaining high transparency.	Struggles with high-dimensionality data.	[29-33]
Gradient boosting and AdaBoost	9	Significantly improved accuracy when compared to random forest or decision trees, able to better handle high-dimensionality data.	Less transparent than random forest or decision trees and more complicated to implement.	[34-41]
Convolutional neural networks	6	Increased accuracy, able to handle high-dimensionality data.	Lacks transparency ("black-box"), difficult to implement and computationally expensive, requires a large data set, ideally suited to pose estimation, which has not been applied extensively in the literature.	[11,43-47]
Long short-term memory-based neural networks	1	Accurate and able to handle high-dimensionality data, excellent for time series data.	Lacks transparency ("black-box"), difficult to implement and computationally expensive, requires a large data set, may not be suited to all data sets.	[19]
Deep Gaussian covariance networks	1	Leverages neural networks to train parameters for Gaussian covariance functions.	Lacks strong real world validation in the literature, suffers from the same general drawbacks as other neural networks.	[48]
Radial basis function	2	May provide improved accuracy, relatively simple architecture.	Lacks strong real world validation in the literature, suffers from the same general drawbacks as other neural networks	[49,50]
Fuzzy and grey neural networks	2	Potential solution to handling high degrees of uncertainty and variability inherent to sports data.	Lacks strong real world validation in the literature, suffers from the same general drawbacks as other neural networks	[51,52]

Discussion

K-nearest neighbor has some practical limitations to the sample sizes it can efficiently analyze. However, its simplicity and versatility are clear. Integration of special sensors allowing for more precise data collection has improved KNN injury recognition models and increased their ability to identify factors that contribute to injury. Enhanced identification of predictive injury features at the resolution of an individual athlete allows coaches and medical personnel to alter training methods to avoid the identified injury risk. However, KNN has been relegated to the role of comparison algorithm in many of the papers discussed in this article. This should not dissuade future researchers from considering it for use, though.

Another simple algorithm, K-means lends itself well to feature extraction. Based on the recent work in the literature, K-means can be used to classify biokinetic data. Alternatively, K-means can effectively be used to predict future high-performing players. However, a more interesting application may be found in the preprocessing of data. K-means clustering may be applied to data sets early in the exploration phase, rather than as a final predictive algorithm. In any case, K-means should be considered when possible.

Support vector machines can be used to both predict the occurrence of an injury and elucidate the risk factors that contribute to injury. However, in the recent literature, SVM-based models have met with mixed success. Even so, SVMs should be considered when predicting sports injury events, especially when dealing with high-dimensionality data. Notably, the best performing SVM models are built as ensemble models, combining the advantages of several algorithms.

Decision trees may also be suitable in medical decision making as they provide reasonable classification accuracy combined with simple representation of gathered knowledge. More importantly, they provide a remarkably transparent decision-making process, allowing deep exploration of features. And, due to this transparency, the decision-making process can be easily validated by an expert that greatly enhances its utility in situations containing high uncertainty. Random forest models increase predictive accuracy compared to decision trees at the expense of reduced transparency. Additionally, they may struggle when data contains high dimensionality, though condensing may provide adequate abatement. Even with the stated limitations, both decision tree and random forest models have performed reasonably well in specific situations and their application should be considered.

Gradient boosting and AdaBoost represent significant improvements in predictive capabilities over classic regression as well as the decision trees on which they are based. They are easier to implement and are more transparent than neural networks while possessing a capacity for large feature sets. Additionally, they are particularly useful when applied in the context of injury prediction where classification can be limited to a binary choice. In cases where transparency is less critical than predictive accuracy, gradient boosting provides a balance between complexity and performance.

While gradient boosting provides various advantages over simpler models, neural networks tend to be the most accurate and powerful ML algorithms currently available. This performance comes at the price of increased complexity, training time, data requirements, and computational resources. Despite these drawbacks, papers rank CNNs, recurrent neural networks (RNNs), and other NN architectures favorably against comparison algorithms. However, there is a lack of robust real-world validation largely due to the lack of readily available large data sets. Researchers are also using player-mounted sensors to collect raw time series data. While this is a valid approach to data collection, it fails to make use of the powerful image recognition and pose-estimation potential of CNNs and limits player enthusiasm for data collection in real-world scenarios. There is a clear route to explore more novel approaches to data collection and structuring, as well as to develop robust studies using real-world data. Any given model architecture or combination of architectures could be applied to any given properly tuned data set. This knowledge alone is of little practical value; however, it demonstrates the need for larger sets of real-world data to further triage algorithm utility between situations. Even with the stated limitations, if the data and computational resources are available, neural networks should be heavily considered.

To illustrate one final observation, it is worth examining a recent systematic review by Bullock et al. The review in question presented 30 studies applying ML to sports injury prediction. Notable in their selection criteria was the inclusion of logistic and Poisson regression, both valid but dated approaches to predictive analysis, as well as the exclusion of novel methodologies for modeling. In fact, 22 of the 30 papers included logistic regression, and 2 of the remaining 8 used Poisson regression [3]. We believe this succinctly illustrates a major bottleneck in the application of ML to sports medicine. A significant number of quality studies are failing to make full use of modern, powerful ML algorithms. Instead, they rely on well-studied but potentially inadequate regression techniques, in addition to falling prey to some other pitfalls discussed earlier. Recent research that does attempt to move past these relatively simple models often fails to produce reliable, generalizable results. Additionally, these papers are often of limited value to those looking for practical applications of ML. Despite these drawbacks, we feel that it is unreasonable to dismiss the usefulness or real-world applicability of ML based on decidedly outdated methodologies.

Limitations

Many of the articles examining neural networks proposed a novel algorithm but validated on a small, artificial data set. Without transparent, real-world data or clear explanations of the proposed data collection and preparation, they do not provide concrete information on algorithm efficacy. Additionally, while most articles detail the equations used, many do not explicitly present the model structure, nor do they provide code.

Problems with data and algorithm transparency are not limited to neural network-focused papers. Many of the other papers discussed in this review rely on small or artificial data sets. Additionally, there is a lack of consistent validation techniques and a large potential for mishandling of data. Notably, there exists a persistent problem with multicollinearity in physiological data sets that was rarely addressed in the literature.

Inter-article variability in algorithm efficacy may also prevent strong conclusions from being drawn based on this report. It is difficult to compare the absolute performance of algorithms presented in two or more papers unless they are tested in the same way on identical data sets. Most papers do not provide the requisite information to make such direct comparisons.

## Conclusions

The continued implementation of machine learning to sports injury prediction faces several challenges. There exists a lack of uniform data sets related to sports injury, resulting in an inability to easily test and validate novel approaches to modeling. Furthermore, that data is being collected inefficiently, particularly with respect to the use of cumbersome player-worn sensors. Model performance is difficult to compare due to the individualized nature of ML model architectures and a lack of transparent reporting regarding algorithm construction. In some cases, outdated or inappropriate models are being applied for the sake of ease of implementation. Logistic regression is often considered an ML algorithm due to its ability to produce a categorical output, but it is not adaptive like other ML techniques and is consistently outperformed by modern ML algorithms. Surprisingly, logistic regression models continue to be used as a prediction tool, often with poor performance. Many injury prediction studies rely entirely on these older techniques, resulting in the conclusion that ML is of little clinical use.

One potential solution to the aforementioned issues is the creation of open-source, uniform data sets that can be tailored to the strengths of targeted algorithms. The vast amounts of data available to sports teams and sports casting agencies, notably, high-quality video footage, could be used to generate large databases for the training of pose-estimation-based CNNs. This would provide researchers with a large, reliable, uniform data set with which to train and validate. It would also eliminate the need to collect data using unreliable athlete-worn sensors. An additional benefit of pose-estimation-based prediction is the generalizability that will likely result, allowing pre-trained networks to be tuned to multiple sports with relative ease. Despite the outlined challenges, significant potential exists within this space. By thoughtfully selecting algorithms and by building adequate data sets, researchers will be able to explore more novel approaches and continue to push the boundaries of ML capability in improving sports medicine outcomes.
